# How much time is available for antenatal care consultations? Assessment of the quality of care in rural Tanzania

**DOI:** 10.1186/1471-2393-11-64

**Published:** 2011-09-24

**Authors:** Moke Magoma, Jennifer Requejo, Mario Merialdi, Oona MR Campbell, Simon Cousens, Veronique Filippi

**Affiliations:** 1 Department of Obstetrics & Gynaecology, Bugando Medical Centre and Teaching Hospital P.O.BOX 1370, Mwanza, Tanzania; 2 Institute for International Programs, Johns Hopkins Bloomberg School of Public Health, Baltimore, MD 21205, USA; 3 Department of Reproductive Health and Research, World Health Organization, Geneva Switzerland; 4 Department of Infectious Diseases Epidemiology, London School of Hygiene and Tropical Medicine, Keppel Street, London WC1E 7HT, UK

## Abstract

**Background:**

Many women in Sub-Saharan African countries do not receive key recommended interventions during routine antenatal care (ANC) including information on pregnancy, related complications, and importance of skilled delivery attendance. We undertook a process evaluation of a successful cluster randomized trial testing the effectiveness of birth plans in increasing utilization of skilled delivery and postnatal care in Ngorongoro district, rural Tanzania, to document the time spent by health care providers on providing the recommended components of ANC.

**Methods:**

The study was conducted in 16 health units (eight units in each arm of the trial). We observed, timed, and audio-recorded ANC consultations to assess the total time providers spent with each woman and the time spent for the delivery of each component of care. T-test statistics were used to compare the total time and time spent for the various components of ANC in the two arms of the trial. We also identified the topics discussed during the counselling and health education sessions, and examined the quality of the provider-woman interaction.

**Results:**

The mean total duration for initial ANC consultations was 40.1 minutes (range 33-47) in the intervention arm versus 19.9 (range 12-32) in the control arm p < 0.0001. Except for drug administration, which was the same in both arms of the trial, the time spent on each component of care was also greater in the intervention health units. Similar trends were observed for subsequent ANC consultations. Birth plans were always discussed in the intervention health units. Counselling on HIV/AIDS was also prioritized, especially in the control health units. Most other recommended topics (e.g. danger signs during pregnancy) were rarely discussed.

**Conclusion:**

Although the implementation of birth plans in the intervention health units improved provider-women dialogue on skilled delivery attendance, most recommended topics critical to improving maternal and newborn survival were rarely covered.

## Background

Antenatal care (ANC) visits provide an opportunity to reach pregnant women with important preventive and treatment interventions as well as counselling on a variety of topics such as birth and complication readiness and the importance of skilled delivery and immediate postnatal care [1]. An additional benefit of regular ANC visits includes the development of a strong provider-woman relationship that can result in improved obstetric outcomes[2].

The amount of time spent on health education, advice and counselling during ANC consultations is key to the effectiveness of ANC in improving health behaviours and care seeking during pregnancy, labour and delivery and in the immediate postpartum period [3]. The information provided during ANC visits enables women and their family members to recognize and act on danger signs or complications, and adopt health promoting behaviours such as adhering to prescribed treatments and referral advice [2,4-6].

The scope of health education and counselling on pregnancy and related complications provided to women during ANC visits in most Sub-Saharan African countries where over half of all maternal deaths occur, however, is often inadequate or nonexistent[3,7]. This may be a contributing factor to the discrepant pattern of high ANC but low skilled birth attendance (SBA) uptake in many of these settings.

Maternal mortality is very high in Tanzania, estimated at 454 deaths/100,000 live births[8]. Ninety-six percent of pregnant women seek ANC from a skilled provider at least once and approximately 43% attend four times or more. Coverage of skilled delivery care is 50%, but notable urban/rural disparities in coverage persist (83.1% in urban versus 42.3% in rural)[8]. Approximately 65% of women do not receive any postnatal care, and only about 31% receive immediate postnatal care (within two days of delivery)[8]. The median gestational age at first ANC attendance is around five and a half months. Although counselling and health education on various aspects of reproductive and child health services are recognized as essential health interventions[9], only 47% of pregnant women in the country receive information from providers on danger signs in pregnancy[10]. A recent study in a rural district found that only about 25% of women attending ANC were informed of danger signs in pregnancy and labour, suggesting that coverage of health education and counselling services may be considerably lower in some regions of the country[11]. Available evidence also suggests that information on the importance of immediate postnatal care on maternal and early neonatal health is rarely provided during antenatal consultations[12].

The focused ANC package based on the WHO model was introduced in Tanzania in 2002,[13] although it was not implemented in some districts until several years later. The package recommends that women with uncomplicated pregnancies should attend ANC four times with the first visit before 16 weeks followed by subsequent visits at 20-24 weeks, 28-32 weeks and the fourth visit at 36 weeks[13]. Health education and counselling are included as essential components of care in the guidelines for each visit. However, the time allotted to counselling has been reported to be minimal in some parts of the country[14].

Studies on the content of ANC provided to women are needed in low-resource countries to improve the quality of ANC. Although some information on the quality of ANC services is collected through national surveys such as Demographic and Health (DHS) and health service assessment, detailed data on counselling, health education or promotion of skilled delivery and postnatal care are often missing.

This paper reports the scope and quality of ANC services provided to women in Ngorongoro, a rural district in northern Tanzania. It compares time spent for various components of ANC and quality of care provided in the control and intervention arms of a cluster randomized controlled trial (RCT) that aimed at examining the effectiveness of birth plans in improving utilization of skilled delivery and postnatal care (Magoma et al, submitted). Although a component of the recommended focused ANC in Tanzania, birth plans are rarely implemented during routine ANC consultations in the study district[12].

## Methods

### Study sites and population

Sixteen dispensaries offering maternal and child health (MCH) services in Ngorongoro district, rural northern Tanzania were included in the study. According to the 2002 Tanzania national census, the total district population was 129,362 of which 29,489 were women in the reproductive age group[15]. Given the crude birth rate of 44.8 per 1,000 population for rural Tanzania[10], it is estimated that there are approximately 6,300 deliveries each year. ANC attendance is high (over 90%), and initial attendance is at 24 weeks gestation on average. Counselling during ANC is provided both individually and in groups, and is more often on HIV/AIDS than on any other topic[12].Far fewer women utilize the available health units for delivery. Coverage of skilled birth attendance is estimated to be as low as 7% (Johnson et, 2005 unpublished with permission)[10].

### Study description

The study was descriptive and used mixed methods of data collection. Quantitative methods were combined with direct observation of ANC consultations. For the purposes of the cluster randomized control trial, the 16 participating health units were randomly grouped into either the intervention arm where birth plans were introduced or the control arm where the standard of ANC as per district protocol was offered (eight health units in each arm). Antenatal and post natal care services were generally available at all clinics (Monday to Friday, 7:30 am - 3:30 pm). Providers worked more than eight hours a day in some clinics because women would frequently arrive close to the end of official hours. Clinic attendees included a mixture of women seeking antenatal and postnatal care.

Between October-December 2008, providers in the intervention units were trained for a total of two half working days at a training workshop, followed by a second training for two half working days at their respective health units. The training covered the implementation of birth plans during ANC and the importance of involving male partners or others identified by women as participants in their care. Providers were also instructed to encourage women to bring their male partners for future ANC visits if they did not accompany them at the time of study recruitment. Major components of the birth plans included: 1) plans for a place of delivery (preferred place identified, transport arrangements made), 2) calculation of the expected costs and money saving strategies for delivery, 3) plans for identifying someone to accompany the woman to the delivery site and another to look after her household, 4) plans for identifying possible blood donors, and 5) plans for addressing any complications arising during pregnancy, labour, delivery, and in the postnatal period (for mother and newborn), and 6) strategies for overcoming any other barriers to accessing skilled delivery care. One copy of the birth plans was retained by the woman so that she could present it to providers at the delivery site, and a second copy was kept at the health unit.

Providers' performances during practical training sessions were timed and recorded on a video camera and later jointly reviewed by trainers and providers. Follow-up training to re-enforce skills learned was conducted at each health unit on clinic days.

Providers in the control units were trained for one half working day at a workshop in the concept of the study in their arm followed by another one half working day in their respective health units supervised by MM. Topics covered included general concepts of focused ANC, and collection and recording of study-related data from study participants and participants' follow-up. Health care providers in both arms of the study were also informed of the process evaluation during the training sessions and before data collection commenced.

Providers in both the intervention and control arms of the study had previously undergone basic and in-depth training on the concepts and implementation of focused ANC by the district MCH team and various NGOs involved in MCH care. All had at least one year experience in implementing focused ANC.

### Data collection

The process evaluation was conducted over a period of approximately ten weeks from January to March 2009 at all 16 health units and took place during the implementation of the cluster randomized trial. ANC consultations were observed, recorded using a digital voice recorder, and timed using a stopwatch. The total time spent on the consultation and on each component of care delivered was recorded.

A health unit assessment form was used to collect information on clinic characteristics, provider adherence to the focused ANC model in all clinics, and on provider implementation of the birth plans in the intervention clinics. Some of the questions in the assessment form were adapted from the Tanzania Service Provision Assessment Survey, 2006 questionnaire[16], and the Tanzania 2004-06 DHS survey questionnaire.

The health units were visited unannounced. Each unit was visited once or twice for observation of the ANC consultations and once for postnatal care. Information was collected on a maximum of five consultations during any single visit to a health unit. Systematic random sampling was used to select up to five consultations depending upon the volume of patient flow at the health unit. The data collected were reviewed with providers after health unit working hours.

**Figure 1:**
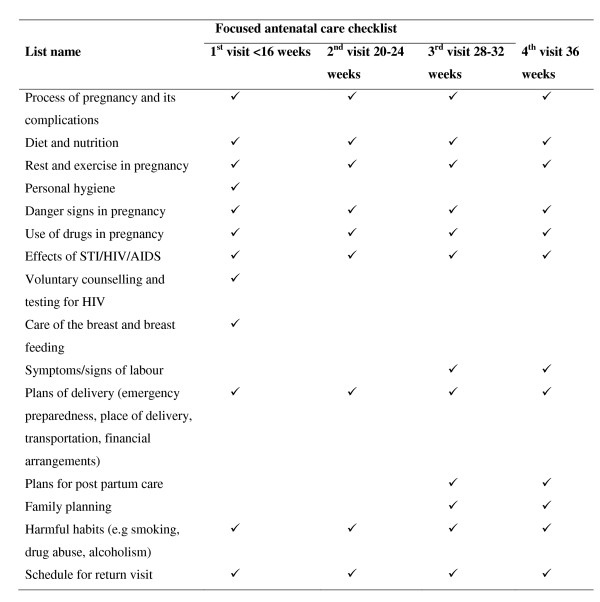
**Focused antenatal care checklist for client education and counselling in Tanzania**.

Health care providers were asked to hang a digital voice recorder around their necks to record the entire consultation. This strategy was used to protect the woman's privacy during physical examinations. After seeking consent from both care providers and women, MM and one trained assistant, both familiar with Kiswahili and the two major local languages (Ma and Kitemi) observed the consultations except the physical examinations. The total duration of the consultations and the time allotted for delivering each component of care was recorded on the health unit assessment form. The components of care assessed included: history taking, examination for maternal and foetal well being, drug administration, and counselling and health education. Counselling and health education included time spent explaining the importance of skilled delivery care for all women, voluntary counselling and testing (VCT) for HIV, health education for HIV/AIDS, birth plans and other topics as stipulated in the focused ANC guidelines for Tanzania (Figure 1). At the time of the study, health units were using the opt-out strategy for VCT for HIV/AIDS. Because only one health unit had reagents for routine syphilis screening, this service was excluded from the assessment.

On the same day of the visit or immediately after leaving the health unit, MM recorded the observations of the quality of provider-client interaction findings in a diary. Information on the availability of essential supplies (e.g. drugs and equipment) and other items needed to support for the delivery of quality ANC was collected.

### Data analysis

The recorded information was analyzed by the principal investigator (MM) assisted by an experienced MCH nurse who reached consensus on the amount of time allotted to each component of care and on the quality of care delivered.

The average total time spent for antenatal consultations for each clinic was calculated by dividing the sum of the time for each component of care delivered divided by the number of consultations assessed to obtain a cluster level summary that was entered in the health unit assessment form. Cluster level summaries on the time spent for providing specific components of care were also calculated. The summaries were later used to calculate the time spent for total consultation in each arm of the study as well as for specific components of ANC.

The quality of the provider-client interaction was ranked good if the provider allowed women to ask questions and responded to their questions. The quality was ranked fair if the provider allowed women to ask questions but did not respond to their concerns nor try to ascertain if the women understood what was discussed. The discussions were ranked bad if the provider neither allowed women to ask questions, nor tried to determine if women understood what they were being told. The interactions were categorized as undetermined if for any reason the ANC sessions were too short to allow assessment of the provider-women interaction. A health unit was categorized to have "good" provider-client interaction if all consultations in the particular health unit were ranked fair or good.

Quantitative data was entered into SPSS statistical package (version 16.0) and later transferred into STATA (version 9) statistical package for cleaning and analysis. The average time for initial and revisit ANC (2nd visit or higher), and postnatal care consultations were calculated and compared between the two study arms using unpaired t-test statistic. Times for components of ANC were similarly calculated.

### Ethical considerations

Ethical approval was obtained from the Institutional Review Boards of the World Health Organization, London School of Hygiene and Tropical Medicine, and the National Institute for Medical Research in Tanzania. Permission was also granted from Arusha region and Ngorongoro district administrative and health authorities and staff at participating health units. Informed verbal consent was obtained from all providers and women who participated in the process evaluation.

## Results

A total of 23 service providers were observed (11 in the intervention arm and 12 in the control): nursing officers (2), midwives (5), MCH nurses (8), nurse auxiliaries (4) and clinical officers (4).

73 initial ANC consultations were observed (36 in the intervention arm and 37 in the control). A total of 35 re-visit ANC consultations were observed (18 in the intervention and 17 in the control). Since not all women were given drugs or immunized at each ANC consultation, a total of 20 consultations were observed for this component of care (11 in the intervention health units versus 9 in the control). The reported average number of pregnant women seen per working day in each clinic did not differ between the two study arms (mean 3.5 95% CI 2.2-4.8 for the intervention versus 5.0 95% CI 2.8-7.3 for the control arm p-value = 0.2072).

### Time for initial, subsequent ANC and postnatal care consultations

Table 1 shows the time spent on the initial ANC consultations. On average, providers in the intervention units spent twice as much time as providers in the control units for the total consultation, spending longer on specific components of care including counselling and health education, and examination. Providers in the intervention units spent slightly more time for history taking than in the control arm (4.4 [95% CI 3.3, 5.5] versus 3.1 [95% CI 2.2, 4.1] p = 0.0596). There was no evidence that the time spent on drug administration and immunization differed between the two arms of the study.

**Table 1:** Duration for initial antenatal consultation in the birth plan intervention in Ngorongoro, rural Tanzania

Time spent	Intervention (n = 8)			Control (n = 8)			p-value
		
	Mean	Minimum	Maximum	Mean	Minimum	Maximum	
Average total time in minutes for consultation	40.1[36.0-44.3]	33	47	19.9[14.5-25.3]	12	32	<0.0001
Average time in minutes for history taking	4.4[3.3-5.5]	3	7	3.1[2.2-4.1]	2	5	0.0596
Average time in minutes for health education and counselling	24.5[20.7-28.3]	19	32	10.5[7.1-13.9]	5	18	<0.0001
Average time in minutes for examination	5.0[3.5-6.5]	3	8	2.5[1.9-3.1]	2	4	0.0047
Average time in minutes for drug administration	6.1[3.9-8.4]	3	10	5.3[4.7-5.8]	4	6	0.4006

Note: Unless otherwise stated numbers indicate the mean cluster level time in minutes and those in brackets indicate respective 95% confidence intervals. The p-values are from the t-test statistic for the mean difference between the two arms of the study.

Table 2 shows the time for the subsequent antenatal consultations and reveals a similar pattern to that observed of the initial consultations.

**Table 2:** Duration for subsequent antenatal consultations in the birth plan intervention in Ngorongoro, rural Tanzania

Time spent	Intervention (n = 8)			Control (n = 8)			p-value
		
	Mean	Minimum	Maximum	Mean	Minimum	Maximum	
Average total time in minutes for ANC consultation	23.3[19.3-27.3]	15	31	10.3[7.1-13.9]	6	17	0.0001
Average time in minutes for familiarization or history taking	2.9[1.8-3.9]	2	5	1.5[1.1-1.9]	1	2	0.0176
Time in minutes for health education and counselling	13.8[10.6-16.9]	6	17	4.5[1.1-7.3]	0	10	0.0001
Time in minutes for examination	4.0[2.3-5.7]	2	8	2.0[0.9-3.1]	1	5	0.0355
Time in minutes for drug administration	2.7[2.3-3.1]	2	3	2.8[2.0-3.5]	1	4	0.070

Note: Unless otherwise stated numbers indicate the mean cluster level time in minutes and those in brackets indicate respective 95% confidence intervals. The p-values are from the t-test statistic for the mean difference between the two arms of the study.

### Scope of health education and counselling

Providers in all intervention health units consistently counselled women on the importance of delivering at the available health units when helping them formulate their birth plans. In contrast, providers in only one control health unit briefly counselled women on skilled delivery care (information not shown). Providers in both the intervention and control health units consistently provided VCT for HIV/AIDS during initial ANC visits, and blood test results were communicated to the women before they left the clinics. Some aspects of prevention of maternal to child transmission of HIV (PMTCT) were also discussed during all ANC consultations.

Tanzania's focused ANC guidelines recommend that women make plans for postpartum care during the third visit (at 28-32 weeks gestation). The importance of postnatal care was consistently emphasized in 5 out of the 8 health units in the intervention arm of the study. It was not consistently emphasized in three of the intervention health units because women typically initiated ANC late. Consequently, there was insufficient time to cover this topic along with all other recommended topics. In contrast, women in only one control health unit were informed about postnatal care and the amount of information provided was brief and inconsistent across consultations.

All health units in the intervention arm and all except one in the control arm received a good score for provider-client interaction. Women asked questions freely and even participated in some health unit activities like weighing other women and recording this information on the ANC cards. Providers in all health units spoke the local language and needed interpreters only occasionally.

### Assessment of the availability of items to support quality ANC services

All health units experienced stock-outs of drugs and equipment needed for the delivery of routine ANC (Table 3). For example, only five out of the eight health units in each arm had a well functioning blood pressure machine, and Aldomet, a widely used first line drug for treating non severe high blood pressure in pregnancy in Tanzania, was only available in one health unit. Although described as a rare occurrence by providers and the district health office, most health units lacked iron tablets.

**Table 3:** Availability of items to support quality ANC services in the two arms of the trial

Essential	Intervention (n = 8)	Control (n = 8)
Privacy during ANC consultations*	8	8
Well functioning blood pressure measuring machine	5	5
Foetalscope	8	8
Iron tablets	3	3
Folic acid tablets	6	8
Antihelminth drug for de-worming women	8	8
At least one broad spectrum antibiotic	8	8
Aldomet tablets	1	0
Antimalarial for intermittent presumptive malaria treatment	8	8
At least one drug for the treatment of Chylamydia, Gonorrhoea or Syphilis	8	8

Note: *A room that ensured visual and auditory privacy during ANC individual counselling/health education or examination.

Numbers indicate number of health units in the respective study arm which had the specified items.

## Discussion

The implementation of the birth plan intervention significantly improved the total time for consultations and for most components of ANC including health promotion and counselling. The increased time spent on counselling in the intervention units suggests that training of providers on birth plans can translate into measurable improvements in provider practices.

The study did not improve the quality of care as measured by provider-patient interactions since consultations in both arms of the study were generally rated as "good". This is a somewhat surprising finding because a logical assumption is that spending more time with women should translate into improved provider-patient communication between providers and clients. The fact that most providers were able to speak the local languages, were willing to work past regular hours to accommodate women's needs, had served in the health units for sufficient time to build a sense of trust with the surrounding communities, and knew most of the women attending their clinics, however, suggests that the rapport between providers and patients in the study health units was strong prior to the implementation of the intervention. Our findings are consistent with results from a previous study in an urban setting in Tanzania[17].

The average time for the initial ANC consultation of 40 minutes in the intervention health units compares favourably with WHO recommendations [18] but is shorter than the average duration reported in studies in Southern Tanzania and Thailand (46 minutes and 1 hour, respectively) [14,19]. Similarly, the average time for second and higher consultations in the intervention arm compares favourably with the 20 minutes recommended in the WHO ANC model[18,19]. Providers in the control units fell far short of recommended average time for the first and subsequent visits at the cost of skipping some important topics of recommended care.

Training of providers on birth plans alone did not result in significant changes in the implementation of other aspects of the focused ANC model. Providers need more training on adequately delivering all components of the model, appropriate supportive supervision and regular evaluation of their performances to improve the quality of care. Arguably, the full implementation of the Tanzania's focused ANC model in health units with heavy workloads might require an increase in the number of providers or office hours to cope with increased time requirements to deliver ANC. The re-organization of health unit activities enabling MCH providers more time and resources to provide quality ANC services may be a pragmatic interim solution. Staffing each health unit with an MCH provider responsible for delivering only clinical services could be a long term objective.

This study found that counselling in both control and intervention units was not provided on many topics recommended in Tanzania's national essential health intervention package[9] and focused antenatal care guidelines[13]. Our finding is consistent with evidence from previous studies suggesting that health education and counselling is the least likely component of ANC to be implemented effectively [14,20,21]. Prevailing factors at each health unit such as provider competency with counselling on all recommended topics, attitude towards discussing these topics with women and their families, and workload might have played a role in determining the scope and quality of the counselling sessions. According to both care providers and the district MCH coordinator, previous training on the focused ANC model was not followed-up by supportive supervision and monitoring. This may have contributed to poor motivation to implement the learned skills[22]. Although lack of counselling skills could be another explanation for the observed limited amount of counselling provided in control units, this is unlikely given that providers were able to effectively counsel women on VCT and PMTCT. HIV/AIDS programs in Tanzania are largely supported by international agencies and are usually well supervised and include regular training[5]. Good supervision and performance evaluation by managers could help improve provider competency in delivering health education and counselling services during routine ANC. Including a greater focus on health education and counselling in midwifery and nursing training curricula could be a long term solution to improving the quality of ANC services in the study setting. The lack of emphasis on health education on other topics aside from skilled delivery care and PMTCT indicates that future interventions aimed at improving the quality of care need to emphasize the importance of discussing all recommended health education topics with women during ANC consultations.

The time spent for counselling in this study in relationship to client flow raises some questions about the time providers will need to spend to cover all recommended topics. If more time than is currently spent is needed to deliver all components of recommended care, this may present a challenge for providing individualized counselling in clinics with heavy workloads. Although group counselling for some topics such as danger signs in pregnancy, labour, delivery and postpartum may be an option, other topics such as birth plans may not be amenable to group discussion. Women's lack of autonomy and decision making ability in the study setting plus the sensitivity of some issues (e.g., HIV, sexually transmitted diseases, violence against women) also mean that a group approach cannot completely replace the need for individualized counselling[12].

This study showed that some clinics experienced stock-outs of essential drugs and equipment needed to provide routine ANC. For example, blood pressure was not routinely checked among women in health units which lacked functioning blood pressure machines, and those with elevated levels could be missed with serious consequences to both the women and their unborn babies. Such stock-outs may contribute to women's perceptions of services at health clinics in the district to be of poor quality and discourage them from utilizing available services for delivery and emergency care. If women choose to bypass the health clinics for hospitals to receive skilled delivery care, they may not reach them fast enough because the hospitals are located far from most women's residences[12].

Process evaluation is a useful technique for understanding how well programmes are being implemented, and can be useful for identifying factors that contribute to or detract from smooth implementation[23,24]. This study showed that the implementation of the birth plans intervention was successful, but that providers need more training and support to implement other aspects of the focused ANC model. In addition, the study highlights the gaps in the delivery of routine ANC in the study setting and how these gaps can be addressed to realize the full potential of focused ANC.

The process evaluation had some limitations. The evaluation was not designed to be a comprehensive assessment of the quality of ANC care in the study setting, but focused on evaluating the implementation of the counselling component of routine ANC. The Hawthorne effect-the possibility that some providers modified their behaviour by spending more time with their clients than they would have done in the evaluator's absence may have influenced the study findings given that providers in both study arms knew that they were being assessed. In a study on the quality of ANC in Tanzania, Boller et al 2003 found that providers delivered free ANC services to women who were usually told to pay for some services in the absence of the researchers[17]. In contrast, in a study in West central Tanzania, providers at one hospital abused women even in the presence of a researcher [25], indicating that entrenched habits may not be greatly influenced by outside observers [26]. The researchers who undertook the observations in this study were well known to the care providers, and the possibility that the latter modified their behaviours to conform to the observers' expectation cannot be ruled out.

Ngorongoro is a remote district and consists of a predominantly pastoralist population. Care seeking behaviours in the district are likely to differ from other districts in Tanzania. The relatively low volume of women attending ANC clinics on a given day also allows providers time to give individualized attention to women. This type of individualized care may not be possible in other settings where the number of ANC attendees is higher. The methodological approach used did not allow for blinding of the principal investigator regarding which health units were in the intervention or control arm. His interpretation of the recorded findings might have been influenced by the knowledge of the arm of the study to which the health units were allocated, thereby introducing bias. To reduce this risk, the recorded materials were reviewed by an experienced MCH nurse who was not aware of the health units' allocation to the two study arms, and the final interpretation of the recorded material depended on consensus of the two investigators. The process evaluation commenced a month after the trial implementation and continued for only three months. Repeat assessment at a later date would help determine if results can be sustained in the longer term.

## Conclusion

Most topics recommended in the ANC guidelines are not routinely discussed during ANC consultations in the study setting. Provider competency and willingness to implement all components of Tanzania's focused ANC model including all recommended health education/promotion topics needs further examination. The need for more training, supportive supervision and monitoring should be addressed to improve the quality of care. The limited time allocated for providing the various components of the focused ANC model in the control arm; the fact that not all recommended health education and counselling topics are being discussed despite prior training on the focused ANC model; and the lack of emphasis on explaining to women the importance of early postnatal care are missed opportunities to realize the full potential of antenatal care to improve obstetrical outcomes in the study setting.

## Competing interests

The authors declare that they have no competing interests.

## Authors' contributions

MM designed the study, collected and analyzed the data, drafted the initial manuscript and reviewed subsequent drafts. JR participated designing the study, developing the data collection tools and reviewing the draft manuscripts at all stages. VF, OMRC and SC participated in designing the study, developing the data collection tools and reviewing the initial and final manuscripts. All authors approved the final version of the manuscript.

## Pre-publication history

The pre-publication history for this paper can be accessed here:

http://www.biomedcentral.com/1471-2393/11/64/prepub
